# Maternal and perinatal outcomes of minimally invasive fetal surgeries: experience from two reference centers in Rio de Janeiro, Brazil

**DOI:** 10.1590/1516-3180.2023.0159.R1.16022024

**Published:** 2024-06-17

**Authors:** Luísa Moreira de Ávila, Paulo Roberto Nassar de Carvalho, Renato Augusto Moreira de Sá, Saint Clair Gomes, Edward Araujo

**Affiliations:** I Post-graduate Student. Department of Fetal Medicine, Instituto Fernandes Figueira, Fundação Oswaldo Cruz (FIOCRUZ), Rio de Janeiro (RJ), Brazil.; II Professor, Strictu Sensu Post-graduation, Department of Fetal Medicine, Instituto Fernandes Figueira, Fundação Oswaldo Cruz (FIOCRUZ), Rio de Janeiro (RJ), Brazil.; III Research in Public Health. Department of Fetal Medicine, Instituto Fernandes Figueira, Fundação Oswaldo Cruz (FIOCRUZ), Rio de Janeiro (RJ), Brazil.; IV Research in Public Health. Department of Fetal Medicine, Instituto Fernandes Figueira, Fundação Oswaldo Cruz (FIOCRUZ), Rio de Janeiro (RJ), Brazil.; V Associate Professor, Department of Obstetrics, Escola Paulista de Medicina, Universidade Federal de São Paulo (EPM-UNIFESP), São Paulo (SP), Brazil.

**Keywords:** Fetus, Surgical Procedures, Operative, Perinatal Mortality, Maternal-Child Health Centers, Fetal Surgery, Perinatal Outcomes, Maternal Outcomes, Reference Centers

## Abstract

**BACKGROUND:**

Concerns regarding high open surgery-related maternal morbidity have led to improvements in minimally invasive fetal surgeries.

**OBJECTIVE:**

To analyze the perinatal and maternal outcomes of minimally invasive fetal surgery performed in Rio de Janeiro, Brazil.

**DESIGN AND SETTING:**

Retrospective cohort study conducted in two tertiary reference centers.

**METHODS:**

This retrospective descriptive study was conducted using medical records from 2011 to 2019. The outcomes included maternal and pregnancy complications, neonatal morbidity, and mortality from the intrauterine period to hospital discharge.

**RESULTS:**

Fifty mothers and 70 fetuses were included in this study. The pathologies included twin-twin transfusion syndrome, congenital diaphragmatic hernia, myelomeningocele, lower urinary tract obstruction, pleural effusion, congenital upper airway obstruction syndrome, and amniotic band syndrome. Regarding maternal complications, 8% had anesthetic complications, 12% had infectious complications, and 6% required blood transfusions. The mean gestational age at surgery was 25 weeks, the mean gestational age at delivery was 33 weeks, 83% of fetuses undergoing surgery were born alive, and 69% were discharged from the neonatal intensive care unit.

**CONCLUSION:**

Despite the small sample size, we demonstrated that minimally invasive fetal surgeries are safe for pregnant women. Perinatal mortality and prematurity rates in this study were comparable to those previously. Prematurity remains the most significant problem associated with fetal surgery.

## INTRODUCTION

Fetal surgery involves heterogeneous interventions, varying from simple to complex, in which the primary goal is to improve the health of newborns diagnosed with abnormalities during prenatal period through intrauterine treatments that decrease the morbidity and mortality rates of potentially severe or lethal congenital anomalies.^1–3^

Fetal therapy has seen significant advancements in recent decades, and ultrasound has made the approach to fetal procedures safer owing to the availability of real-time guidance.^1,2,4^ In the early 1980s, open surgical treatments were started ^5,6^, but concerns about the high maternal morbidity rates related to open surgeries have led to the search for less invasive alternatives.^7^ The increasing popularity of videoendoscopic surgery in the 1990s, combined with recent experience in fetoscopy, introduced the concept of endoscopic or minimally invasive fetal surgery ^4^, which is under continuous development and improvement.^8^

In Brazil, one of the first fetal endoscopic surgeries was fetoscopy for the laser treatment of twin-twin transfusion syndrome (TTTS), which was performed in 2001.^9,10^ Since then, several surgical techniques for fetal surgeries, both minimally invasive and open surgeries, specifically for myelomeningocele, have been performed.^10^ Currently, few groups have performed percutaneous endoscopic fetal surgery for the treatment of myelomeningocele; Brazilian and German researchers have pioneered advances in performing this technique. The neurological results of this technique are similar to those of the open technique.^3,10-14^

## OBJECTIVE

This article describes maternal and perinatal outcomes of minimally invasive fetal surgeries performed at two fetal medicine referral centers in Rio de Janeiro, Brazil.

## METHODS

This retrospective study included all pregnant women who underwent delivery and minimally invasive fetal surgery between 2011 and 2019 at the Instituto Fernandes Figueira/Fiocruz (IFF/Fiocruz) and Clínica Perinatal, which are both referral centers for maternal-fetal medicine in Rio de Janeiro, Brazil. Clinical data and outcomes were exclusively assessed by reviewing medical records from the prenatal period to hospital discharge. This study was approved by the local institutional ethics committee under number 27452719.5.0000.5269, in accordance with the National Health Council resolution 466/12.

Outcomes included maternal and obstetric complications, neonatal morbidity, and fetal and neonatal mortality from the intrauterine period to hospital discharge. Maternal complications included anesthetic complications, infectious complications, need for blood transfusion, and admission to intensive care unit. Preterm labor, preterm delivery, chorioamnionitis, and preterm premature rupture of ovular membranes (PPROM) were considered pregnancy complications. Neonatal morbidities were defined as findings of brain injury on ultrasonography, retinopathy of prematurity (ROP), bronchopulmonary dysplasia (BPD), necrotizing enterocolitis (NEC), neonatal infection, need for ventilatory support, admission to the neonatal intensive care unit (NICU), or length of stay in the NICU.

Cerebral injuries were detected using cranial ultrasound in neonates, and they were classified as mild to severe intraventricular hemorrhages ^15^ or as mild to severe periventricular leukomalacia.^16^ ROP was classified into five stages based on the International Classification of Retinopathy of Prematurity.^17^ BPD was defined for newborns under 32 weeks of oxygen therapy > 21% for at least 28 days after 36 weeks of post-menstrual age or discharge to home, whichever came first, or for newborns aged ≥ 32 weeks, oxygen therapy for > 28 days but < 56 days postnatal age or discharge to home, whichever came first.^18^ NEC diagnosis was based on clinical signs and symptoms and radiological findings, surgically confirmed in some cases.^19^ Neonatal infections confirmed via laboratory testing were considered owing to the difficulty of classifying neonatal sepsis based on medical records.

Statistical analyses were performed using SPSS software version 17.0 for Windows (SPSS Inc., Chicago, IL, USA). Frequency measures were used for categorical variables and means and standard deviations were used for numerical variables.

## RESULTS

Seventy-five surgeries were performed between 2011 and 2019. Patients who were followed up and gave birth in the research units were included, totaling 50 patients (8 at IFF/Fiocruz and 42 at Perinatal). Twenty patients had multiple pregnancies; the total of fetus was 70. Pathologies seen were as follows: 18 (36%) patients with TTTS, with or without fetal growth restriction; 12 (24%) with congenital diaphragmatic hernia (CDH); 9 (18%) with myelomeningocele (MMC); 4 (8%) with lower urinary tract obstruction; 4 (8%) with pleural effusion; 2 (4%) with congenital high airway obstruction syndrome (CHAOS); and 1 (2%) with amniotic band syndrome. The outcomes of all pathologies are described together, and for discussion purposes, the pathologies with the highest incidence were analyzed separately (TTTS, CDH, and MMC). One case each of CHAOS and CDH occurred in a woman with multiple pregnancies. Table 1 presents the baseline characteristics of the study population.

**Table 1:** Baseline characteristics of the study population

Maternal variables			Results (N = 50)
Mean age (years)			33 (20 to 42)
**Comorbidities – no. (%)**			
	No		36 (72)
	Yes		14 (28)
		Hypothyroidism	5 (10)
		Diabetes mellitus	1 (2)
		Arterial hypertension	1 (2)
		Thrombophilia	2 (4)
		Others	5 (10)
**Conception – no. (%)**			
	Spontaneous		46 (92)
	Assisted Reproduction Techniques		4 (8)
**Number of fetuses – no. (%)**			
	Singleton pregnancy		30 (60)
	Multiple Pregnancy		20 (40)
**Delivery – no. (%)**			
	Caesarean section		45 (90)
	Vaginal delivery		5 (10)
**Fetal Pathology – no. (%)**			
	Twin-twin Transfusion Syndrome*		18 (36)
	Congenital Diaphragmatic Hernia†		12 (24)
	Myelomeningocele		9 (18)
	Lower Urinary Tract Obstruction		4 (8)
	Pleural Effusion		4 (8)
	Congenital Upper Airway Obstruction Syndrome†		2 (4)
	Amniotic Band Syndrome		1 (2)
**Location of placenta – no. (%)**			
	Anterior		21 (42)
	Others		29 (58)

* with or without Fetal Growth Restriction;

† one case in multiple pregnancy

### Surgical variables

The mean gestational age on the day of surgery was 25 weeks (range, 16–32 weeks), being 21 weeks (17–25 weeks) in the TTTS treatment group; 28 weeks (23–30 weeks) in fetuses with CDH for fetoscopic endoluminal tracheal occlusion (FETO); and 27 weeks (25–28 weeks) in the MMC correction group. 21 (42%) patients received local anesthesia or sedation, 11 (22%) received general anesthesia, and 30 (60%) received spinal anesthesia. The mean surgical time was 80 min (63, 66, and 148 min for TTTS, FETO, and MMC corrections, respectively). 41 patients (82%) received antibiotic prophylaxis, 36 (72%) received tocolytic agents, and 24 (48%) received atosiban. In addition, 21 (42%) patients required more than one surgical procedure: five patients had TTTS; 11 patients had CDH; 6 other patients had other pathologies that were not described in this article. In patients with CDH, the second procedure involves balloon removal through fetoscopy at approximately 34 weeks of gestation. Table 2 presents these data in detail.

**Table 2:** Fetal surgical variables

Surgery variables		Total (N = 50)	TTTS (N = 18)	CDH (N = 12)	MMC (N = 9)
**Anesthesia – no. (%)**					
	Local anesthesia or sedation	21 (42)	9 (50)	6 (50)	-
	General anesthesia	11 (22)	1 (6)	-	9 (100)
	Spinal anesthesia	30 (60)	14 (78)	12 (100)	-
**Antibiotic prophylaxis – no. (%)**		41 (82)	15 (83)	11 (92)	9 (100)
**Tocolytics – no. (%)**		36 (72)	14 (78)	9 (75)	9 (100)
	Atosiban	24 (48)	12 (67)	5 (42)	3 (33)
	Nifedipine	9 (18)	5 (28)	4 (33)	-
	Indomethacin	7 (14)	-	-	7 (78)
**Mean gestational age (weeks)**		25 (16 to 32)	21 (17 to 25)	28 (23 to 30)	27 (25 to 28)
**Surgery time (minutes)**		80	63	66	148
		(30 to 203)	(40 to 120)	(30 to 130)	(110 to 203)
**More than one procedure – no. (%)**		21 (42)	5 (28)	11 (92)]	

TTTS: twin-twin transfusion syndrome; CHD: congenital diaphragmatic hernia; MMC: myelomeningocele

### Maternal complications

Concerning maternal complications, four (8%) patients had anesthetic complications, three (6%) had post-spinal anesthesia headaches, and one (2%) had decreased oxygen saturation and required macronebulization, which quickly improved their clinical condition. Six (12%) patients had infectious complications or sepsis: two (4%) were due to urinary tract infection treated with antibiotics, two (4%) were diagnosed with appendicitis, and two (4%) with chorioamnionitis. Three (6%) patients required blood transfusions and three (6%) had other complications, such as migraine, hypotension, and chest pain. Table 3 lists the maternal variables according to pathology.

**Table 3:** Maternal complications due to fetal surgery

Maternal Complications		Total (N = 50)	TTTS (N = 18)	CDH (N = 12)	MMC (N = 9)
**Anesthetic – no. (%)**		4 (8)	2 (11)	-	1 (11)
	Post-spinal anesthesia headaches	3 (6)	2 (11)	-	-
	Decreased oxygen saturation needing macronebulization	1 (2)	-	-	1 (11)
**Infectious/sepsis – no. (%)**		6 (12)	3 (17)	-	3 (33)
	Urinary tract infection treated with antibiotics	2 (4)	2 (11)	-	-
	Appendicitis	2 (4)	1 (6)	-	1 (11)
	Chorioamnionitis	2 (4)	-		2 (22)
**Required blood transfusion – no. (%)**		3 (6)	2 (11)	-	1 (11)
**Others – no. (%)**		3 (6)	-	2† ‡(17)	1ƾ (11)

† hypotension,

‡ chest pain and

ƾ migraine

TTTS: twin-twin transfusion syndrome; CHD: congenital diaphragmatic hernia; MMC: myelomeningocele

### Antepartum complications

Complications before delivery were classified into intraoperative and postoperative categories based on timing. Among 11 patients (22%), intraoperative issues were noted, with bleeding being the most common (12%), followed by procedure failure (6%), placental abruption (2%), and amniotic detachment (2%). Notably, no intraoperative complications occurred in the TTTS group, while the FETO group experienced one case (8%) of placental abruption on the day of balloon removal, while at MMC group, four cases (45%) of intraoperative bleeding and one case (11%) of amniotic detachment. Postoperative complications were prevalent, affecting 36 patients (72%), with the most frequent being PPROM (40%), followed by preterm labor (26%), intrauterine death (14%), and chorioamnionitis (6%). Antepartum complications varied across groups: the TTTS group had 15 cases (83%), including PPROM (17%), preterm labor (28%), and intrauterine complications (27%); the CDH group experienced 8 cases (67%), primarily PPROM (33%) and preterm labor (17%); and the MMC group saw 8 cases (89%) of antepartum complications, predominantly PPROM (89%). Additionally, chorioamnionitis affected 22% of MMC patients, while preterm labor and appendicitis each occurred in 11% of cases. Table 4 describes the complications according to the frequency of each pathology.

**Table 4:** Antepartum complications due to fetal surgery

Antepartum Complications			Total (N = 50)	TTTS (N = 18)	CDH (N = 12)	MMC (N = 9)
**Intraoperative complications – no. (%)**			11 (22)	-	1 (8)	5 (56)
	Bleeding		6 (12)	-	-	4 (45)
	Amniotic detachment		1 (2)	-	-	1 (11)
	Placental abruption		1 (2)	-	1 (8)	-
	Intraoperative fetal death		-	-	-	-
	Failure to perform the procedure		3 (6)	-	-	-
**Antepartum complications – no. (%)**						
	Yes		36 (72)	15 (83)	8 (67)	8 (89)
		Preterm PROM	20 (40)	3 (17)	4 (33)	8 (89)
		Chorioamnionitis	3 (6)	-	-	2 (22)
		Preterm labor	13 (26)	5 (28)	2 (17)	1 (11)
		Intrauterine death	10/70† (14)	10/37‡(27)	-	-
		Others*	7 (14)	3 (17)	2 (17)	1 (11)
**Mean GA at PROM preterm (weeks)**			31 (18 to 37)	30 (27 to 32)	35 (30 to 37)	29 (26 to 36)
**Mean GA at Chorioamnionitis (weeks)**			32 (18 to 32)	-	-	32 (32 to 32)
**Mean GA at Preterm labor (weeks)**			30 (22 to 36)	29 (22 to 36)	34 (33 to 35)	32 (32 to 32)
**Mean GA at Intrauterine death (weeks)**			22 (18 to 26)	22 (18 to 26)	-	-
**Mean GA at Others** * **(weeks)**			30 (23 to 35)	28 (23 to 31)	35 (35 to 35)	-

* Fetal distress (4), appendicitis (2), and fetal anemia (1).

† The number corresponds to the total number of fetuses, which is 70.

‡ The number corresponds to the total number of fetuses, which is 37.

GA: gestational age; TTTS: twin-twin transfusion syndrome; CHD: congenital diaphragmatic hernia; MMC: myelomeningocele; Preterm PROM: preterm premature rupture of ovular membranes

### Childbirth-related variables

The study encompassed neonatal data across various conditions. On average, gestational age at delivery was 33 weeks, with a range of 25 to 40 weeks, resulting in 83% live births. Most newborns were admitted to the NICU, with 50% requiring resuscitation at the delivery room. Antenatal corticosteroids were administered to 76% of pregnant women. Respirator use was necessary for 48% of newborns, and surfactants were required in 19% cases. Additionally, 24% of newborns needed additional oxygen support after 28 days of life. Notably, mortality was observed in 26% of cases. Patients with TTTS had a mean gestational age at delivery of 31 weeks, with 96% live births. Among CDH patients, the mean gestational age at delivery was 37 weeks, with all infants admitted to the NICU and experiencing respiratory issues. Finally, patients with MMC had a mean gestational age at delivery of 33 weeks, with 100% live births and relatively lower mortality rates. Table 5 describes the baseline characteristics of the study population, while Table 6 summarizes the outcomes of the individual characteristics of each pathology.

**Table 5:** Childbirth-related variables

Childbirth-related variables	Total (N = 70)	TTTS (N = 27)	CDH (N = 12)	MMC (N = 9)
**Mean GA at delivery (weeks)**	33 (25 to 40)	31 (25 to 40)	37 (33 to 39)	33 (28 to 37)
**Born alive – no. (%)**	58 (83)	26 (96)	12 (100)	9 (100)
**Mean birth weight (grams)**	2,045 (500 to 3790)	1,536 (500 to 2830)	2,560 (1700 to 3180)	1,976 (1130 to 3130)
**Mortality**	15 (26)	4 (15)	7 (58)	-
**NICU discharge**	40 (69)	20 (74)	5 (42)	9 (100)
**ICU discharge without sequela no. (%)**	13 (23)	9 (33)	2 (17)	1 (11)
**Number of days in the NICU**	61 (5 to 509)	37 (14 to 509)	45 (29 to 64)	37 (5 to 110)
**Number of hospitalization out of NICU (days)**	88 (2 to 343)	3 (2 to 3)	343 (343 to 343)	-

TTTS: twin-twin transfusion syndrome; CHD: congenital diaphragmatic hernia; MMC: myelomeningocele; GA: gestational age; NICU: neonatal intensive care unit

**Table 6:** Disease-specific variables

Disease-specific variables		Results
**Twin-twin Transfusion Syndrome**		
	Weight discordance before surgery (%)	28 (SD ± 13)
	Quintero Stage - **no. (%)**	
	Stage I	2 (11)
	Stage II	14 (78)
	Stage III	2 (11)
	Stage IV	
	Weight discordance after surgery (%)	27 (SD ± 17)
	Marginal or velamentous cord insertion - **no. (%)**	4 (22)
	Number of anastomoses endoscopically detected (mean)	9 (SD ± 3)
**Congenital Diaphragmatic Hernia**		
	Laterality - **no.** (%)	
	Right	1 (8)
	Left	11 (92)
	Liver Herniation - **no. (%)**	7 (58)
	Lung-head ratio before surgery	0.88 (SD ± 0.13)
	Mean GA at balloon removal (weeks)	34 (33 to 32)
	Need for ECMO **- no. (%)**	2 (17)
	Fetoscopic balloon removal failure needing ultrasound-guided punctures to removal **- no.** (%)	2 (17)
**Myelomeningocele - no. (%)**		
	Type 2 Arnold Chiari syndrome	6 (67)
	Intact suture at birth	6 (67)
	Foot deformity	2 (22)
	Tetraventricular dilatation	4 (45)

GA: gestational age; ECMO: extracorporeal membrane oxygenation

## DISCUSSION

## MMC

The results of the Management of Myelomeningocele Study (MOMS), a randomized controlled trial, seem to have changed the timing of open spina bifida repair from the postnatal to the prenatal period. The trial was interrupted due to better efficacy in patients who underwent prenatal repair than in those who underwent postnatal repair.^3,20^ However, because of the open nature of this fetal surgery, these favorable outcomes occur at the expense of increased risks of uterine dehiscence and rupture, as well as other morbidities in mothers.^3^ As open fetal surgery by hysterotomy is an invasive procedure, fetoscopic surgical techniques aim to minimize surgical trauma.^13^ Meanwhile, following significant technical improvements and a steep learning curve for the surgical teams, fetoscopic fetal surgery is considered on par with open techniques by some researchers, although controlled head-to-head comparisons remain lacking.^3,21,22^

There are few maternal complications related to fetoscopic surgeries for myelomeningocele correction, justifying the continued investment in studies focusing on this technique, which is expected to be disseminated more widely. Regarding maternal complications in our study, two (22%) patients were diagnosed with chorioamnionitis who were treated with intravenous antibiotics, and one patient (11%) required blood transfusion during pregnancy, which was not directly related to surgery but rather to the context of appendicitis during pregnancy.

In a study intended only to assess maternal complications published by Kohl et al.^23^ there was no need for maternal blood transfusion, placental abruption, or spontaneous postoperative uterine contractions at immediate perioperative period; however, they described pulmonary edema in one (1.9%) patient. Nonetheless, the incidence of acute pulmonary edema described in MOMS is 6% ^20^, indicating a lower risk of this complication with fetoscopic surgery. There were no cases of acute pulmonary edema in our study; however, this could have been due to the low number of cases.

Kohl et al.^23^ also described the use of tocolysis for 24 h after the procedure: in two (4%) cases of chorioamniotic detachment after the procedure and in four (7.8%) patients who developed chorioamnionitis.^23^ In our study, all patients also underwent tocolysis for a short time, and we had one (11%) case of chorioamniotic detachment.

Despite the use of tocolysis, premature births remain problematic. Mean gestational age at delivery in our study was 33 weeks, with PPROM occurring in eight (89%) patients, which was the same gestational age described by Diehl et al.^13^ in an observational study with data from 72 patients, and the same as described by Lapa et al.^3^ Lapa et al.^3^ described the rate of PPROM as 80%, like ours. However, in a study published in 2021, which analyzed 170 pregnant women in eight reference centers worldwide, mean gestational age at delivery was 34.5 weeks with a PPROM rate of 67%, which decreased to 38% after modifying the technique used during the study with CO_2_ humidification during fetoscopy.^12^ Furthermore, three neonatal deaths related to prematurity were also reported ^12^, whereas in our study, there were no neonatal or intrauterine deaths related to intrauterine treatment for MMC.

## CDH

Prenatally diagnosed CDH is associated with a high postnatal mortality rate owing to the coexistence of other major defects or various combinations of pulmonary hypoplasia and persistent pulmonary hypertension.^24-29^ Several observational studies have shown that FETO is associated with increased survival among children with severe pulmonary hypoplasia due to isolated left CDH; however, randomized clinical trials have not yet been published.^30^ The TOTAL trial was the first randomized to assess FETO, as this study was terminated early because of identification of benefits of surgery versus expectant management. From 80 patients analyzed, 40 underwent FETO and 40 maintained expectant management.^30^

At surgery group, median gestational age at randomization in the TOTAL trial was 27.7 weeks, and 36 (90%) patients had intrathoracic liver herniation.^30^ In our study, mean gestational age on the day of surgery was 28 weeks, and seven (58%) patients had intrathoracic liver herniation.

Regarding complications described at TOTAL trial related to fetal surgeries, 19 (48%) newborns were diagnosed with PPROM with a median gestational age of 32.5 weeks, one (2%) patient was diagnosed with placental abruption, one (2%) with bleeding when introducing trocater for fetoscopy, and eight (22%) with chorioamniotic detachments.^30^ In our study, four (33%) patients were diagnosed with PPROM, which occurred with a mean gestational age of 35 weeks. There were no cases of bleeding related to introduction of trocaters during fetoscopy or diagnoses of chorioamniotic detachment after fetoscopy, but one (8%) patient was diagnosed with placental abruption after fetoscopy was employed to attempt to remove the balloon. In this case, fetoscopy to remove the balloon was unsuccessful, and it was necessary to puncture the balloon guided by ultrasound after birth. We observed one (8%) case in which fetoscopy to remove the balloon was unsuccessful, and patient developed premature labor and fetal distress after having undergone a cesarean section, which showed the presence of a hemoamnion. In this case, balloon was removed postnatally using ultrasound-guided puncture without complications. At TOTAL trial, there was one (2%) death due to failure to remove the balloon in a patient who did not follow the recommendations and moved to a place far from the trial reference centers.^30^ No fetal deaths were related to balloon removal.

Mean gestational age at delivery in our population was 37 weeks, and all fetuses who underwent the procedure were born alive, with a mean birth weight of 2,560 g. Two (17%) required extracorporeal membrane oxygenation and five (42%) were discharged from the NICU. At TOTAL trial, mean gestational age at delivery was 34.6 weeks, all fetuses who underwent the procedure were born alive, mean birth weight was 2,300 g, and two (5%) required extracorporeal membrane oxygenation. Thirty (75%) patients at TOTAL trial gave birth before 37 weeks, whereas in our study, only two (17%) patients had preterm labor.

Regarding neonatal complications, at TOTAL trial, 12 (75%) newborns were diagnosed with BPD, 1 (6%) had leukomalacia, and 10 (62%) sepsis.^30^ In our study, two (17%) infants were diagnosed with BPD, although we had seven (58%) newborns who needed oxygen after 28 days of life, which suggests that there may have been underdiagnosed in this classification. We did not diagnose leukomalacia in our study, and nine (75%) newborns were diagnosed with neonatal infection. We also obtained two (17%) diagnoses of intracranial hemorrhage and did not have any cases of ROP or NEC.

A multicenter study evaluated 210 pregnancies with CDH treated with FETO performed at a median gestational age of 27.1 weeks, with a median duration of 10 min. PPROM occurred in 47.1% of the patients, and delivery occurred at a median gestational age of 35.3 weeks. In 97.1% of the cases, newborns were live born, and 48% were discharged from the hospital alive.^27^

## TTTS

The first randomized trial conducted to evaluate TTTS' treatment with endoscopic laser surgery compared to serial amnioreduction's treatment was published in 2004 by the Eurofoetus group. The study was terminated early after demonstrating the benefit of the group that underwent laser coagulation of placental anastomoses. In total, 72 patients were included in the laser group. According to Quintero's classification, there were six (8%) patients with stage I, 31 (43%) with stage II, 34 (47%) with stage III, and one (1%) with stage IV.^31^ In our study, two (11%) patients were at Quintero stage I, 14 (78%) in stage II, and two in stage III (11%). None of the patients were in stage IV.

At Eurofoetus study, mean gestational age at delivery at laser group was 33.3 weeks, with a mean birth weight of 1,757 g.^31^ Compared to our study, mean gestational age at delivery was 31 weeks, with a mean birth weight of 1,536 g, which were similar to the results published by Gheorghe et al.^32^, Malshe et al.^33^, and Habli et al.^34^

Regarding complications, at Eurofoetus study, 10 (15%) patients were diagnosed with PPROM within 28 days of the procedure and 16 (12%) fetuses were diagnosed with intrauterine death within 7 days of the procedure; in 76% of patients, at least one fetus survived.^31^ Malshe et al.^33^ described PPROM in 32 (15.8%) cases and fetuses were born alive in 78.3% of cases. Complications in our study were observed in three (17%) patients with PPROM, and there were 10 (27%) intrauterine deaths during pregnancy, which is consistent with the results of the studies cited above.^31,33^ We also had three (17%) patients who experienced other complications, including one case of fetal distress, one case of fetal anemia, as well as a pregnant woman with appendicitis a few weeks after fetoscopy.

Neonatal complications were also described by Eurofoetus group: a total of 12 (8%) newborns died during the neonatal period, intraventricular hemorrhage grade III or IV was noted in two (1%) newborns, and leukomalacia in eight (6%) newborns^31^. In our group, four (15%) newborns were diagnosed with intracranial hemorrhage, but we included mild cases, not just the most severe ones; 20 (74%) newborns were discharged from NICU, with four (15%) deaths registered at neonatal period. Records of patients with leukomalacia were not available. NEC was noted in our study in four (15%) cases, which was a much higher incidence than that described by Gheorghe et al.^32^ (1.8%). Malshe et al.^33^ described 12 (18.5%) cases of placental abruption and four (6.2%) cases of chorioamnionitis^33^. We did not observe chorioamnionitis or placental abruption in pregnancies subjected to laser treatment for TTTS.

At Eurofoetus study, no pregnant woman died or required blood transfusion or hospitalization in the maternal ICU.^31^ No deaths were observed in our study; however, three (17%) patients had infectious complications not directly related to fetoscopy, two (11%) had urinary tract infections, and one (6%) had abdominal sepsis due to appendicitis.

A retrospective study published by Habli et al.^34^ evaluated 152 pregnant women who underwent laser fetoscopy to treat TTTS and the incidence of postoperative complications; 147 (97%) patients in this study underwent procedure using epidural anesthesia, while the most used anesthesia was spinal in 14 (78%). In the same study, it was impossible to complete the procedure in five patients: two cases due to peritoneal leaks and three cases due to intraamniotic bleeding from trocar introduction or laceration of the chorion plate by the laser.^34^ No complications were observed in our surgeries.

## CONCLUSION

Our study assessed fetal surgeries performed at two reference centers for fetal medicine in Rio de Janeiro, Brazil. Fetal surgeries in Brazil play an important role, mainly because there is no possibility of pregnancy termination due to abortion.

Our study has some limitations, such as its retrospective descriptive nature based on data from medical records and the small sample size when considering the time evaluated. Analysis of less common diseases, combined with a small sample size selected for convenience through patients referred directly to the two reference centers, suggests that there is a possible selection bias, which means that our results cannot be extrapolated to other populations.

Therefore, intrauterine surgery may improve the prognosis of these fetuses; however, we cannot ignore the fact that intrauterine fetal surgeries, even minimally invasive surgeries, can result in complications in pregnant women and are associated with a higher risk of prematurity. Nonetheless, based on our findings, we conclude that intrauterine fetal surgeries are safe for pregnant women, with low morbidity, perinatal mortality, and prematurity rates, comparable to those previously reported. Premature birth remains a major problem associated with fetal surgery.
